# Efficacy and safety of manual acupuncture manipulations with different frequencies on epigastric pain syndrome (EPS) in functional dyspepsia (FD) patients: study protocol for a randomized controlled trial

**DOI:** 10.1186/s13063-017-1845-3

**Published:** 2017-03-06

**Authors:** Shou-hai Hong, Sha-sha Ding, Fei Wu, Ying Bi, Fu Xu, Yi-jia Wan, Li-hua Xuan

**Affiliations:** 1 Acupuncture Department, Zhejiang Provincial Hospital of TCM, Hangzhou, China; 2 Rehabilitation Department, Tianjin Nankai Hospital, Tianjin, China; 3 Traditional Chinese Medicine Department, The First Hospital of Wuhu city, Wuhu, Anhui China

**Keywords:** Functional dyspepsia, Epigastric pain syndrome, Manual acupuncture, Lifting-inserting, Manipulations

## Abstract

**Background:**

Manual acupuncture (MA) manipulations are one of the key factors influencing acupuncture effects in traditional Chinese medicine theory. Different MA manipulations contain different stimulating parameters, thus generating different acupuncture responses or effects. Evidence has demonstrated that acupuncture is effective for functional dyspepsia (FD). However, the effects of different stimulating parameters of MA manipulations on FD remain unclear.

**Methods/design:**

This study is a randomized controlled trial with a four-arm, parallel-group structure. Patients with FD with epigastric pain syndrome (EPS) will be included and randomly allocated into four groups: three MA manipulation groups (separately treated with a frequency of 1 Hz, 2 Hz, or 3 Hz) and a control group. All groups will receive omeprazole as a basic treatment and acupuncture: in the MA manipulation groups, the needles will be manipulated manually with three different frequencies on the basis when *de qi* is reached, while in the control group, the needles will be inserted without any manipulation. All patients will receive acupuncture treatment of five consecutive sessions per week for 2 weeks and be followed up at 4, 8, and 12 weeks. The primary outcomes of the study include patients’ response to the treatment. The secondary outcomes include dyspeptic symptoms, quality of life, mental status, fasting serum gastrin, motilin, and ghrelin concentrations, and adverse events. The protocol was approved by the Ethics committee of the First Affiliated Hospital of Zhejiang Chinese Medical University (2016-K-057-01).

**Discussion:**

The aim of this study is to evaluate the efficacy and safety of MA manipulations with different stimulating parameters (different frequencies) on EPS in patients with FD.

**Trial registration:**

Chinese Clinical Trial Registry, ChiCTR-IOR-16008189. Registered on 30 March 2016.

**Electronic supplementary material:**

The online version of this article (doi:10.1186/s13063-017-1845-3) contains supplementary material, which is available to authorized users.

## Background

### Introduction

Functional dyspepsia (FD) is a common disease causing upper abdominal symptoms occurring in the absence of organic disease that is likely to explain them. According to the Rome III criteria, FD is divided into two subgroups: epigastric pain syndrome (EPS) and postprandial distress syndrome (PDS) [1]. FD has negative impacts on health and also results in a high economic burden [2]. Standard management of FD has not yet been established, and satisfactory medications are also unavailable [3]. The management of FD remains a major issue in health clinics.

In recent years, evidence-based medicine has demonstrated that acupuncture is beneficial for relieving dyspepsia symptoms and associated negative emotions, thus improving patient quality of life [4–6]. The acupoints and acupuncture stimulus such as manual acupuncture (MA) manipulations are the key elements inducing the effect. Related research studies on treating FD with acupuncture usually focus on the specificity or compatibility of acupoints [6, 7]. However, the MA manipulations to treat FD have not been fully investigated, causing difficulties in the establishment and application of the best acupuncture treatment for FD. Researchers had showed that different MA manipulations contain different stimulating parameters, thus generating different acupuncture responses or effects [8–10]. Our previous study also demonstrated that lifting-inserting MA manipulations with a frequency of 2 or 3 Hz could better inhibit acute visceral nociception in rats with gastric distension than manipulations with a frequency of 0.5 or 1 Hz [11]. Therefore, we assume that the effects of MA manipulations with different stimulating parameters on EPS in FD are different. The aim of this study is to evaluate the different efficacies and safety of lifting-inserting MA manipulations with different frequencies on EPS in patients with FD.

### Hypothesis

Based on our previous study, our hypothesis is that MA manipulations with different frequencies have different effects on dyspepsia symptoms and quality of life, and that the high frequency will have a better effect than the low frequency.

### Objectives

The trial objectives are to clarify the different therapeutic effects and possible biological mechanisms of lifting-inserting MA manipulations with different frequencies on EPS in patients with FD.

## Methods/design

### Design

**Fig. 1:**
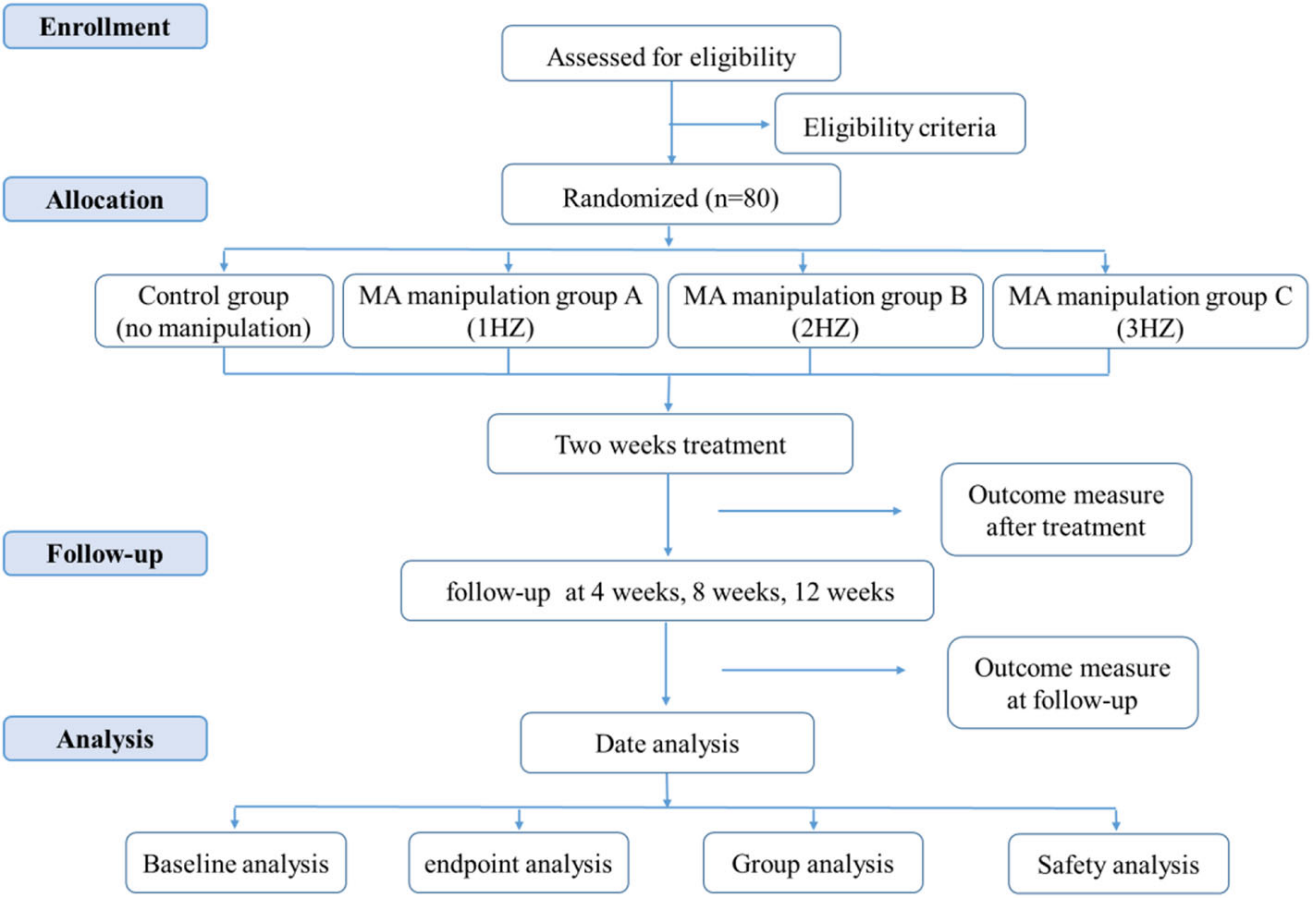
Flow diagram of the study

A four-armed parallel randomized controlled trial will be conducted at the Department of Acupuncture and Moxibustion, Zhejiang Provincial Hospital of TCM. The flow diagram of the entire trial is shown in Fig. 1. The Standard Protocol Items: Recommendations for Interventional Trials (SPIRIT) 2013 checklist is given in Additional file 1.

### Participants

#### Inclusion criteria

Participants will be included if they: (1) meet the Rome III FD criteria [1] (the new diagnostic standard for FD) and have clinical symptoms of epigastric pain syndrome (EPS); (2) are between the ages of 18 and 60 years; (3) have received no other treatments related to the gastrointestinal system 1 week before enrollment and during the study; (4) are willing to agree with the study protocol and sign a written informed consent.

#### Exclusion criteria

Participants who present any of the following criteria will be excluded: (1) serious structural disease (disease of heart, lung, liver, kidney, digestive system, or hematopoietic system) or mental illness; (2) other diseases that could interfere with acupuncture treatment, e.g., clotting disorders or leukopenia, active skin infection, pacemaker, epilepsy, or anticoagulant therapy; (3) women who are pregnant or breastfeeding; (4) difficulties in attending the trial (e.g., paralysis, cancer, dementia, drug addiction, time constraints, surgical operation); (5) difficulty being followed up.

### Recruitment, randomization, allocation concealment, and blinding

The participants will be recruited through advertisements in hospital websites and bulletin boards. The participants who meet the inclusion criteria and have signed written informed consents will be randomly assigned to one of three MA manipulation groups (groups A–C) or the control group in a 1:1:1:1 ratio by central randomization performed by an independent statistician from the Tigermed Pharmaceutical Science and Technology Co., Ltd. Random numbers will be generated using the PROC PLAN of SAS 9.2 (SAS Institute Inc., Cary, NC, USA). Opaque sealed envelopes will be used to conceal the group to which participants have been allocated. Investigators who have contact with the participants should be unaware of the random allocation. To preserve masking, only the acupuncturists will have access to the treatment allocation. The participants, recruiters, and outcome assessors (the evaluator and the statistician) will be unaware of the group assignments.

### Intervention

Participants will be randomly allocated into one of four groups: MA manipulation group A, B, C or the control group. Everyone in each group will receive basic treatment for FD (omeprazole, a classic acid suppressing drug, is effective for the treatment of FD; 20 mg of omeprazole 2 × daily, 30 min before meals) and acupuncture after randomization. The only difference in the four groups is the acupuncture manipulation (e different stimulating parameters of the MA manipulations).

#### Control group

The needles will be inserted into the same acupoints and depth as for the MA manipulation groups but without any manipulation. The treatment period or follow-up is also the same as for the MA manipulation groups.

#### MA manipulation groups A, B, and C

The main acupoints ST36, PC6, and RN12 will be needled in each group. Additional acupoints will also be used based on symptom differentiation. For stagnation of liver qi, RN17 and LR13 (bilateral) are added; for deficiency of spleen qi and stomach qi, one adds BL20 (bilateral) and BL21 (bilateral); for stomach disorder due to liver qi, LR14 (bilateral) and LR3 (bilateral) are added; for damp heat in middle Jiao, one adds SP9 (bilateral) and ST44 (bilateral). Acupoints will be localized according to the 2008 World Health Organization standards [12]. Sterilized stainless steel needles (Φ0.25 mm × 25 mm or 0.25 mm × 40 mm, Tianjin Hua Hong Medical Co., Ltd., Tianjin, China) will be used for all acupuncture procedures. The length of the needle will be chosen according to the acupoint.

**Fig. 2:**
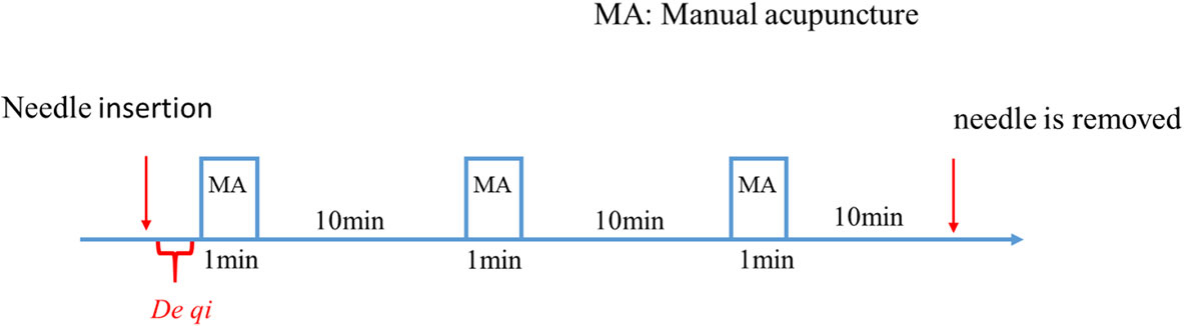
The lifting-inserting MA manipulation procedure

After a *de qi* sensation is achieved, the needles in MA manipulation groups A, B, and C will be respectively manipulated manually with three different frequencies (1, 2, or 3 Hz) of lifting-inserting MA manipulations (see Table 1). The lifting-inserting MA manipulation procedure is shown in Fig. 2. Briefly, after *de qi*, the lifting-inserting MA manipulation will be performed for 1 minute, three times, separated by 10-minute intervals. The entire procedure takes 33 minutes. The acupuncture treatment consists of 10 sessions over a period of 2 weeks (one session per day, five continual sessions per week, with a 2-day interval between the 2 weeks) after randomization. All participants will be followed up three times in 10 weeks after treatment (at 4, 8, and 12 weeks).

**Table 1:** Parameters of three different lifting-inserting MA manipulations in MA manipulation groups

MA manipulation group	Frequency (Hz)	Operation of MA	Depth (mm)	Duration (s)
Group A	1	Neutral reinforcement and reduction	2–3	60
Group B	2			
Group C	3			

*MA* Manual acupuncture

In neutral reinforcement and reduction, the needle is perpendicular to the skin, and the force and amplitude of the lifting-inserting displacement is uniform; the lifting-inserting distance is 2–3 mm

All acupuncture procedures will be performed by the same licensed acupuncturist who has more than 5 years of clinical experience. The acupuncturist will use a metronome to maintain the rhythm. Before applying the needling manipulations, the acupuncturist will repeatedly practice the manipulations on an ATP-II acupuncture manipulation parameter tester (manufactured by Shanghai University of Traditional Chinese Medicine, Shang Xin Medical Technology Company). The ATP-II device is used to measure various manual acupuncture manipulation parameters through electrical resistance-sensing technology. MA manipulation waveforms appear in real time on the computer screen. During the practice phase, the acupuncturist regulates his manipulations according to the waveforms shown on the computer screen until the waveforms became reproducible. This practice ensures that the angle, depth, and frequency of lifting-inserting manipulation are consistent and repeatable.

### Outcomes

**Fig. 3:**
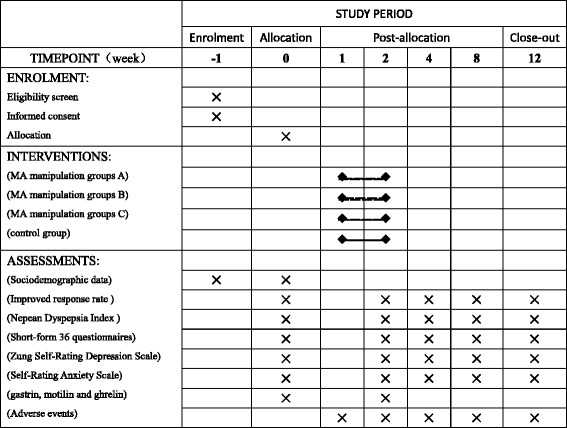
Schedule of enrollment, interventions, and assessments

The primary outcomes of the study include patients’ response to the treatment [13]. The secondary outcomes include dyspeptic symptoms [14], quality of life [15], mental status [16, 17], fasting serum gastrin, motilin, and ghrelin concentrations, and adverse events. Each outcome variable will be assessed before and after treatment, and follow-up will be conducted at 4, 8, and 12 weeks in all groups (Fig. 3).

#### Primary outcomes

Patients’ response to the treatment will be measured by an improved response rate (IRR) [13]: for EPS, the improvement of at least two scores and/or no occurrence of epigastric pain and epigastric burning included in the scale are regarded as positive responses. The Symptom Index for EPS is a 4-indicator scale assessing two main dyspepsia symptoms (epigastric pain and epigastric burning) with a scale ranging from 0 to 3. A score of 0 indicates symptoms not present; 1 indicates occasional and mild symptoms that do not interfere with social activities; 2 indicates prolonged and obvious symptoms interfering with work or rest; and 3 indicates severe symptoms with an impact on work or rest. The IRR is the percentage of patients who are symptom-free or have shown improvement in at least two scores at the end of the treatment and follow-up.

#### Secondary outcomes

Dyspeptic symptoms will be assessed by the Chinese version of the Nepean Dyspepsia Index (NDI) [14], including the intensity, frequency, and level of interference of postprandial fullness, early satiety, epigastric pain, and epigastric burning sensation. The intensity of each symptom is graded and scored as follows: 0, absent; 1, mild; 2, moderate; 3, severe; 4, critical. The frequency of each symptom is also graded: 0, absent; 1, occasionally (1–2 days/week); 2, sometimes (3–5 days/week); 3, frequently (every day, but intermittent symptoms); 4, continuous symptoms. The level of interference of each symptom is scored and graded as follows: 0, none; 1, mild interference; 2, moderate interference; 3, severe interference; 4, critical interference. The number in front of each grading indicates the score of the corresponding symptom; the score for each symptom in the checklist of cardinal dyspeptic symptoms is calculated by adding its scores in the corresponding frequency, severity, and level of discomfort. The dyspeptic symptom sum score (DSSS) is the sum score of the four symptoms in the checklist.

The quality of life will be evaluated by the Short-Form 36 questionnaire (SF-36) [15]. Mental status will be measured by the Zung Self-Rating Depression Scale (SDS) [16] and Self-Rating Anxiety Scale (SAS) [17]. The SF-36 measures quality of life (QoL) across eight domains; the score of each domain = [(actual raw score − lowest possible raw score)/raw score range] × 100. For the SDS score, the following equation is used: SDS Index = Raw Score × 1.25. The grading of the SDS is as follows: SDS Index less than 53 points is considered normal, 53–62 is considered mild depression, 63–72 as moderate depression, and 73 and higher as severe depression. For SAS grading, the following categories are used: normal range (less than 50), mild anxiety (50 to 59), moderate anxiety (60 to 69), and severe anxiety (70 and higher).

Fasting serum levels of gastrin, motilin, and ghrelin will be measured by the enzyme-linked immunosorbent assay. A fasting venous blood sample will be drawn from the basilic vein prior to breakfast early in the morning before and after treatment.

Any adverse reactions of patients will be recorded in the case report form (CRF), including swelling, pain, bruise at the sites of needle insertion, or discomfort, palpitation, dizziness, etc., after acupuncture.

### Sample size

The sample size calculation is based on the results of trials by Li et al. [18] and Ji et al. [19] and the recommendation of acupuncture specialists in China. The mean value of the NDI score in the 2.5 Hz group is 82.28, the standard deviation is 6.97; the mean value of the NDI score in the 2 Hz group is 86.73, the standard deviation is 7.10; the mean value of the NDI score in the 1 Hz group is 76.50, the standard deviation is 8.06; the mean value of the NDI score in the control group is 65.85, the standard deviation is 16.47. To detect a significant difference between any two groups with a power of 90% and a type I error of 5%, the calculated number of patients is 17. Considering a 30% dropout rate, the total sample size required is 88 patients.

### Statistical analysis

The statistical analysis will be performed by two independent statisticians. The statisticians are blinded to treatments and study protocol. Statistical analysis will be conducted on the basis of an intention-to-treat (ITT) analysis. Missing values will be imputed by the last observation carried forward (LOCF) method. Categorical data will be analyzed with the McNemar chi-square test. Continuous data will be analyzed by analysis of variance (ANOVA). If the data trend over time and over time by treatment interactions, the repeated-measures ANOVA will be used. A *P* value less than 0.05 is regarded as statistically significant.

### Quality control and dropout criteria

All researchers will be required to undergo special training, including trial design, patient inclusion and exclusion criteria, and proper completion of the CRF. All practitioners, who have majored in acupuncture and received an acupuncture degree, are qualified doctors of traditional Chinese medicine. Patients who want to stop participating in this study, do not complete the treatment over two visits, violate the study protocol, withdraw consent for participation, or use prohibited treatments for FD will be dropped from the study. The reasons for dropouts and withdrawals from the study will be fully recorded.

## Discussion

This is a protocol for a randomized, double-blind, controlled trial on MA manipulations with different stimulating parameters on EPS in patients with FD. According to the Rome III criteria, FD can be divided into two subgroups: epigastric pain syndrome (EPS) and postprandial distress syndrome (PDS) [1]. The differences in pathological mechanisms between EPS and PDS have been discussed controversially in the past. However, some new research studies have shown that these two subgroups differ in risk factors and pathophysiology, so their pathological mechanisms are different [20, 21]. EPS and PDS should be treated with different approaches [22]. The diagnosis and treatment guidelines of dyspepsia in China also point out that clinical trials should separately study EPS and PDS. At present, acupuncture is widely used to treat disease pain, and acupuncture analgesia has gained worldwide acceptance. Therefore, we have chosen FD patients with EPS for this research.

Creating proper control groups in acupuncture clinical research is a conundrum. Rather than look for a better placebo, we designed the trial so that all patients in each group would receive acupuncture using the same needles, inserted to the same depth at the same acupoints. What differed between the groups is the frequency to which the needles are manipulated. In the control group, the needles will be inserted without any manipulation. The comparison is between four techniques. Because each patient will receive some kind of acupuncture, it is less likely that expectations will form a large part of the therapeutic effect.

Acupuncture has been accepted to effectively treat diseases by the insertion of needles into specific acupoints, and the effect is manifested when the inserted needles are manipulated by hand (manual acupuncture, MA) [23–25]. MA, consisting of needle manipulations by hand, such as lifting, thrusting, twisting, twirling, or other complex combinations, is more traditional and widely used in practice. Lifting-thrusting and twisting-twirling are the most basic needle manipulations in MA. Researchers found that different MA manipulations, containing different stimulation parameters (including frequency, angle, and depth, etc.) [8], could produce different physiological responses [9, 10] and therapeutic effects [26, 27]. Lifting-thrusting needle manipulation was more suitable for the treatment of gastrointestinal movement disorders than twisting-twirling manipulation [28, 29]. In our previous study, we demonstrated that lifting-inserting needle manipulations with a frequency of 2 or 3 Hz could better inhibit acute visceral nociception in rats with gastric distension than manipulations with a frequency of 0.5 or 1 Hz [11]. Therefore, we assume that the effects of lifting-inserting manipulations with different stimulation parameters (different frequencies) on EPS in FD were distinct. In order to illuminate the rule and mechanism of lifting-inserting manipulations with different frequencies treating FD with EPS, we designed the randomized controlled double-blind trial to reveal the difference in the effects of lifting-inserting manipulations with different frequencies. At the same time, we will observe some related hormone concentrations and analyze the relations in effect, hormones, and different manipulations. Thus, we can better optimize and standardize the acupuncture protocols to guide the treatment of FD with EPS.

### Trial status

Participant recruitment is currently ongoing.

## Additional file

Additional file 1: SPIRIT checklist. (DOCX 24 kb)

## Abbreviations

ANOVA: Analysis of variance
CRF: Case report form
DSSS: Dyspeptic symptom sum score
EPS: Epigastric pain syndrome
FD: Functional dyspepsia
IRR: Improved response rate
ITT: Intention to treat
LOCF: Last observation carried forward
MA: Manual acupuncture
NDI: Nepean Dyspepsia Index
QoL: Quality of life
SAS: Self-Rating Anxiety Scale
SDS: Self-Rating Depression Scale
TCM: traditional Chinese medicine
