# Supplementary material for: Efficacy and safety of manual acupuncture manipulations with different frequencies on epigastric pain syndrome (EPS) in functional dyspepsia (FD) patients: study protocol for a randomized controlled trial
Source: Trials. 2017 Mar 6;18:102. doi: 10.1186/s13063-017-1845-3 (PMC5339951; doi:10.1186/s13063-017-1845-3)
Supplement: Additional file 1: — SPIRIT checklist. (DOCX 24 kb) [file 13063_2017_1845_MOESM1_ESM.docx]

| Table 1. SPIRIT 2013 Checklist: Recommended Items to Address in a Clinical Trial Protocol and Related Documents* | | |
| --- | --- | --- |
| Section/Item | Item Number | Description |
|  |  |  |
| Administrative information | | |
| Title | 1 | Efficacy and safety of manual acupuncture manipulations with different frequencies on the epigastric pain syndrome (EPS) in functional dyspepsia (FD) patients: study protocol for a randomized controlled trial |
| Trial registration | 2a | Chinese Clinical Trial Registry: ChiCTR-IOR-16008189. |
|  | 2b | - |
| Protocol version | 3 | - |
| Funding | 4 | This study was supported by the Traditional Chinese medicine science and technology project of Zhejiang Province (grant number 2016ZA810), the National Public Special Fund for Traditional Chinese Medicine-scientific Research (grant number 201507006-01), the National Natural Science Foundation of China (grant number 81603677). |
| Roles and responsibilities | 5a | *SHH, YB, XF, YJW* and *LHX* [Acupuncture Department, Zhejiang Provincial Hospital of TCM, Hangzhou, China], *SSD* [Rehabilitation Department, Tianjin Nankai Hospital, Tianjin, China], *FW*[Traditional Chinese Medicine Department, The First Hospital of Wuhu city, Wuhu, Anhui, China]  **Authors' contributions**  *SHH, SSD, FW, YB, XF, YJW* and *LHX* all contributed to the development of the study protocol. *SHH* and *SSD* drafted the manuscript. All authors read and approved the final manuscript before submission. |
|  | 5b | - |
|  | 5c | - |
|  | 5d | - |
| Introduction |  |  |
| Background and rationale | 6a | Manual acupuncture (MA) manipulations is one of the key factors influencing acupuncture effects in Traditional Chinese Medicine theory. Different MA manipulations contain different stimulating parameters, thus generating different acupuncture response or effect. Evidences demonstrates acupuncture is effective for functional dyspepsia (FD). While the effects of different stimulating parameters of MA manipulations on the FD remains unclear. |
|  | 6b | As our aim is investigate the different effects of different stimulating parameters of MA manipulations, so we designed the control group as follows: the needles will be inserted into the same acupionts and depth as MA manipulations groups but without any manipulation. The treatment period or follow-up is also the same as MA manipulations groups. |
| Objectives | 7 | **Hypothesis**  Based our previous study, our hypothesis is that MA manipulations with different frequencies have different effect on the dyspepsia symptoms and quality of life, in which the high frequency will have better effect compared with the low frequency.  **Objectives**  To clarify the different therapeutic effects and possible biological mechanisms of lifting-inserting MA manipulations with different frequencies on the EPS in FD patients. |
| Trial design | 8 | The trial is designed as a randomised, controlled, participants, recruiters and outcome assessors (the evaluator and the statistician) blinded trial with four parallel groups and a primary endpoint of patients’ response to the treatment. The randomization will be performed as block randomization with a 1:1:1:1 allocation |
| Methods |  |  |
| Participants, interventions, and outcomes | | |
| Study setting | 9 | A four-armed parallel randomized controlled trial will be conducted at the Department of Acupuncture and Moxibustion, Zhejiang Provincial Hospital of TCM. |
| Eligibility criteria | 10 | **Inclusion Criteria**  Participants who meet the following criteria will be included: (1) Meeting the Rome III FD criteria, and with clinical symptoms of epigastric pain syndrome (EPS); (2) Between the ages of 18 and 60 years; (3) Receiving no other treatments related to the gastrointestinal system one week before enrollment and during study; (4) Winging to agree with a study protocol and sign a written informed consent.  **Exclusion Criteria**  Participants who present any of the following criteria will be excluded: (1) With serious structural disease (disease of heart, lung, liver, kidney, digestive system or hematopoietic system) or mental illness; (2) With other diseases that could interfere with acupuncture treatment, e.g., clotting disorders or leukopenia, active skin infection, pace-maker, epilepsy, or anticoagulant therapy; (3) Women who are pregnant or breastfeeding; (4) Having difficulties in attending the trial (e.g., paralysis, cancer, dementia, drug addiction, time constraint, surgical operation); (5) Being not followed up. |
| Interventions | 11a | Participants will be allocated into one of four groups, the MA manipulation group A, B, C, or control groups. Everyone in each group will receive basic treatment for FD (Omeprazole, a classic acid suppressants drug, is effective for the treatment of FD patients. 20 mg of omeprazole 2 × daily, 30 min before meals) and acupuncture. The only differences in the four groups is the manipulation of acupuncture (that means different stimulating parameters of MA manipulations).  Control group: The needles will be inserted into the same acupionts and depth as acupuncture groups but without any manipulation. The treatment period or follow-up is also the same as acupuncture groups.  MA manipulation group A, B and C: Main acupoints ST36, PC6 and RN12 will be needled in each group. Additional acupoints will also be used based on symptoms differentiation. Stagnation of liver qi, add RN17, LR13 (bilateral); deficiency of spleen qi and stomach qi, add BL20(bilateral), BL21(bilateral); stomach disorder due to liver-QI, add LR14(bilateral), LR3(bilateral); damp-heat in middle-Jiao, add SP9(bilateral), ST44(bilateral). Acupoints will be localized according to the 2008 World Health Organization standards[12]. Sterilized stainless steel needles (Φ0.25 mm×25 mm or 0.25 mm×40 mm, Tianjin Hua Hong Medical Co., Ltd., Tianjin, China) will be used for all acupuncture procedures. The length of the needle will be chosen according to the acupoint.  After manipulation until a de qi sensation is achieved, the needles in MA manipulation group A, B and C will be respectively manipulated manually with three different frequencies (1, 2 or 3 Hz) of lifting-inserting MA manipulations (see table 2). The procedure of lifting-inserting MA manipulation see figure 2. Briefly, after de qi, the lifting-inserting MA manipulation will be performed for 1 minute, totally three times with interval of 10 minutes. The procedure will last for 33 minutes. Acupuncture treatment consists of 10 sessions over a period of 2 weeks (one session per day, five continual sessions per week, 2 days interval between 2 weeks). All participants will be followed up for 3 times in 10 weeks after treatment (respectively at 4 weeks, 8 weeks and 12 weeks). |
|  | 11b | - |
|  | 11c | - |
|  | 11d | - |
| Outcomes | 12 | The primary outcomes of the study include patients’response to the treatment. The secondary outcomes include dyspeptic symptoms, quality of life, mental status, fasting serum gastrin, motilin and ghrelin concentration, and adverse events. Each outcome variable will be assessed before and after treatment, and follow-up will be conducted at 4 weeks, 8 weeks and 12 weeks in all groups (see Table 2). |
| Participant timeline | 13 | Acupuncture treatment consists of 10 sessions over a period of 2 weeks (one session per day, five continual sessions per week, 2 days interval between 2 weeks). All participants will be followed up for 10 weeks afterwards. Each outcome variable will be assessed before and after treatment, and follow-up will continue for up to 10 weeks after treatment in all groups |
| Sample size | 14 | Sample size calculation is based on the result of trials by X. J. li[13] and X. Ji[14] and the recommendation of acupuncture specialists in China. The mean value of NDI score in the 2.5Hz group is 82.28, the standard deviation is 6.97; the mean value of NDI score in the 2Hz group is 86.73, the standard deviation is 7.10; the mean value of NDI score in the 1Hz group is 76.50, the standard deviation is 8.06; the mean value of NDI score in the omeprazolec group is 65.85, the standard deviation is 16.47. To detect a significant difference between any two groups with a power of 90% and type I error of 5%, the calculated number of patients is 17. Considering a 30% drop-out rate, the total sample size needs 88 patients. |
| Recruitment | 15 | The participants will be recruited through advertisements in hospital websites and bulletin boards. |
| Assignment of interventions (for controlled trials) | | |
| Allocation |  |  |
| Sequence generation | 16a | The participants meeting the inclusion criteria and signing written informed consents will be randomly assigned to 1 of 3 acupuncture groups (groups A–C) or omeprazole group (control group) in a 1:1:1:1 ratio by central randomization performed by an independent statistician, who come from the Tigermed Pharmaceutical Science And Technology Co., L.. Random numbers will be generated using the PROC PLAN of SAS 9.2 (SAS Institute Inc., Cary, NC, USA). |
| Allocation concealment mechanism | 16b | Opaque sealed envelopes will be used to conceal which group participants have been allocated to. Investigators who have contact with the participants should be unaware of the random allocation. |
| Implementation | 16c | To preserve masking, only the acupuncturists will access to the treatment allocation. The participants, recruiters and outcome assessors (the evaluator and the statistician) will be unaware of study-group assignments. Method of generating the allocation sequence (e.g., computer-generated random numbers), and list of any factors for stratification. To reduce predictability of a random sequence, details of any planned restriction (e.g., blocking) should be provided in a separate document that is unavailable to those who enroll participants or assign interventions. |
| Blinding (masking) | 17a | The participants, recruiters and outcome assessors (the evaluator and the statistician) will be blinded of study-group assignments. |
|  | 17b | - |
| Data collection, management, and analysis | | |
| Data collection methods | 18a | - |
|  | 18b | - |
| Data management | 19 | All researchers will be required to undergo special training, including trial design, patient inclusion and exclusion criteria, and on filling in the CRF. All practitioners, having majored in acupuncture and got an acupuncture degree, are qualified doctors of Traditional Chinese Medicine.  All of the data were filled in electro-CRF twice by different persons, and the difference between the first and second electro-CRF fill-in was checked automatically by computer to maintain accuracy. |
| Statistical methods | 20a | The statistical analysis will be performed by two independent statisticians. The statisticians are blinded to treatments and study protocol. Statistical analysis will be conducted on the basis of intention-to-treat analysis (ITT). Missing values will be imputed by the last observation carried forward (LOCF) method. Categorical data will be analyzed with the McNemar chisquare test. Continuous data will be analyzed by analysis of variance (ANOVA). If the data trends over time and over time by treatment interactions, the repeated-measures ANOVA will be used. A P value less than 0.05 is regarded as statistically significant. |
|  | 20b | - |
|  | 20c | - |
| Monitoring Data monitoring | 21a | - |
|  | 21b | - |
| Harms | 22 | - |
| Auditing | 23 | - |
| Ethics and dissemination |  |  |
| Research ethics approval | 24 | The protocol was approved by the Ethic committee of the First Affiliated Hospital of Zhejiang Chinese Medical University (2016-K-057-01). |
| Protocol amendments | 25 | - |
| Consent or assent | 26a | - |
|  | 26b | - |
| Confidentiality | 27 | - |
| Declaration of interests | 28 | All authors declare that they have no conflict of interests regarding the publication of this paper. |
| Access to data | 29 | - |
| Ancillary and post-trial care | 30 | - |
| Dissemination policy | 31a | We will communicate trial results to participants, health care professionals, the public, and other relevant groups via publication, reporting in results databases, or other data-sharing arrangements, including any publication restrictions. |
|  | 31b | - |
|  | 31c | - |
| Appendices Informed consent materials | 32 | - |
| Biological specimens | 33 | - |
